# Short-term mentalization-based therapy for common childhood mental disorders – a pilot quasi-randomised controlled trial

**DOI:** 10.1177/13591045251316619

**Published:** 2025-01-27

**Authors:** Agneta Thorén, Karin Lindqvist, Julia Pertoft Nemirovski, Jakob Mechler

**Affiliations:** 1 11264 The Erica Foundation, Sweden; 2 Department of Psychology, 7675 Stockholm University, Sweden; 3 Department of Psychology, Uppsala University, Sweden

**Keywords:** Children, mixed disorders, outcome, psychodynamic, short-term, mentalization, parent sessions

## Abstract

Internalizing and externalizing psychiatric disorders among children are common and debilitating, affecting family interactions, learning and peer relations. The aim of the present quasi-randomised pilot-study was to investigate preliminary effects of a mentalization-based time-limited treatment (MBT-C) for children with mixed psychiatric disorders. The trial comprised 17 children, aged 4–11 with mixed disorders, and their parents, admitted to an outpatient psychotherapy clinic. Quasi-randomization allocated patients to 12 sessions MBT-C with parallel parent support, or wait-list control. Compared to wait-list controls, significant improvements were observed in child pathology (*d* = 1.23*, p* = .006), therapist-rated global functioning (*d* = 1.73, *p* = .002), parent-perceived overall distress and impairment (*d* = 1.42, *p =* .017), and child-perceived emotional distress (*d =* 1.32, *p =* .024). No significant effects were observed for parent-perceived symptoms (*d* = 0.41, *p* = .28). Within-group changes and long-term effects were calculated for all participants including the wait-list after being crossed-over to treatment. Results were either maintained or further improved at 6- and 12-months follow-ups. This trial provides preliminary support for MBT-C in children with mixed disorders.

## Plain Language Summary

Psychiatric disorders, both internalizing and externalizing, are common and have a significant impact on children’s lives. They affect their family dynamics, learning abilities, and relationships with peers. The purpose of this pilot study was to investigate the effects of a time-limited treatment called mentalization-based therapy for children (MBT-C) who have a variety of psychiatric disorders. The study included 17 children, aged 4–11, who had common mental health problems, along with their parents. These participants received treatment at an outpatient psychotherapy clinic. Patients were assigned to either 12 sessions of MBT-C with additional support for their parents or placed on a wait-list control. The results showed that compared to the wait-list controls, the group receiving MBT-C experienced significant improvements in various areas. These included the child’s overall pathology as rated by a psychiatrist, the child’s global functioning as evaluated by the therapist, the overall distress and impairment experienced by the parents, and the child’s own perception of emotional distress. However, there were no significant effects observed in terms of the parents’ perception of symptoms. The positive results from this trial were maintained or even further improved during the 6- and 12-month follow-up assessments. This study provides preliminary evidence supporting the use of MBT-C as an effective treatment for children with common mental health problems.

## Introduction

Anxiety, depression and behavioural disturbances are common mental health problems in children and adolescents (Weisz & Gray, 2008). Childhood disorders are associated with significant impairments in daily functioning, affecting family interaction, peer relations and learning (Costello et al., 1996). Moreover, these conditions are frequently comorbid, complicating treatment and prognosis (Garber & Weersing, 2010; Kashani & Orvaschel, 1988). Without appropriate intervention, affected children are at risk for escalating problems with long-term individual and societal implications (Goodman et al., 2011).

More than half of pre-school aged children (3–4 years) with high levels of disruptive behavior problems will continue to show these symptoms when in school-age (Campbell, 1995). Notably, over half of adult mental health disorders have their origins in childhood and adolescence (Kessler et al., 2005), underscoring the critical need for early interventions. Identifying effective treatments for children suffering from emotional and behavioural disorders is a growing concern world-wide (US Public Health Service, 2000).

During the last decades, an increase in mental ill-health among children and adolescents has been reported internationally (Collishaw, 2015) as well as in Sweden (National Board of Health and Welfare, 2017). Public mental health services are facing important challenges in providing early interventions and empirically validated treatments, tailored to meet different maturational needs. Ideally, applicable interventions should be cost-effective (i.e., time-limited) and capable of targeting critical developmental mechanisms underlying prevalent childhood dysfunctions in emotional regulation, behavioural patterns, and relational dynamics.

In recent decades, substantial progress has been made in evaluating the effectiveness of treatments for children. Notably, short-term psychodynamic psychotherapy (STPP) with a focused and active therapeutic stance has earned interest for its potential efficacy in treating childhood and adolescent mental disorders (Abbass et al., 2013; Bakermans-Kranenburg et al., 2003; McLaughlin et al., 2013). Previous reviews, such as Bloom (2002), indicate that brief interventions are as effective as their open-ended counterparts for conditions like anxiety, depression, and disruptive behaviour disorders in young people.

The first meta-analysis on brief psychodynamic interventions for children and adolescents, conducted by Abbass et al. (2013), comprised 11 quasi-experimental or randomized controlled trials, with a total of 655 patients. The study found support for the effectiveness of brief psychodynamic interventions across various common mental disorders, with superior effects compared to wait-list, minimal contact or treatment-as-usual, and no differences in effects compared to other active treatments. In an ambitious randomized controlled trial encompassing 470 adolescents with major depressive disorder, Goodyer et al. (2017) found no significant differences in main outcome between brief psychodynamic therapy, cognitive-behavioural therapy, and brief psychosocial intervention.

Despite increased research, the evidence base for child and adolescent interventions remains limited, often constrained by methodological heterogeneity and small samples sizes. Apart from an obvious need for more high-quality studies, there is also a demand for quasi-experimental and one-group pre-post designs conducted in naturalistic settings (Midgley et al., 2021a). Without understanding and addressing real-world factors, such as comorbidity which are often excluded from efficacy trials, the applicability of effective treatments in routine clinical practice will be significantly impeded (Midgley et al., 2021a; Weisz & Gray, 2008). Royal Academy of Sciences (2010) draws special attention to the need of naturalistic studies on young and middle-childhood aged populations (0–10 years), and strongly suggests child-adapted outcome measurements, allowing even young patients to participate in research.

It has lately been suggested that the apparent gap between research and practice in child and adolescent clinical trials may be met by a greater emphasize on transdiagnostic interventions, i.e., targeting the causal and sustaining factors underlying diverse common clinical problems (Midgley, Sprecher et al., 2021b). Transdiagnostic interventions are considered particularly pertinent for elucidating emotional and behavioural problems in children and adolescent (Chu et al., 2016; Harvey, 2013), primarily due to the age groups’ significantly higher prevalence of comorbidity, and variability in prominent symptoms throughout development (Garber & Weersing, 2010). In addition to their suitability for examining key developmental processes across disorders, transdiagnostic interventions hold significant potential for clinical application, as they facilitate the addressing of diverse diagnoses within a unified treatment framework (Midgley, Sprecher et al., 2021b).

Mentalization-Based Treatment (MBT) is such a transdiagnostic evidence-based, short-term intervention developed out of psychodynamic tradition and theory, with theoretical roots also in social cognition, developmental psychology and neuroscience. It was initially developed and evaluated for borderline personality disorder in adults (Bateman & Fonagy, 2016), and has been adapted for adolescents with emotional instability disorders (Rossouw & Fonagy, 2012) and parent-infant work (Midgley & Vrouva, 2012). Mentalizing, or the ability to perceive and understand ourselves and others in terms of subjective states and mental processes, is recognized as a vital skill in social interactions and a core mechanism in the development of emotional and behavioural regulation, and the forming of stable relationships (Fonagy & Allison, 2012; Midgley et al., 2017). Deficits in mentalizing have been linked to a variety of internalizing and externalizing problems in children (Halfon & Bulut, 2017; Sharp & Venta, 2012), but also with less sensitive parenting (Zeegers et al., 2017) and lack of parental locus of control in parent-child interactions (Groh et al., 2017).

However, despite its conceptual roots in developmental theory, less concern has been paid to the development of mentalization-based treatments for middle-childhood. To fill this gap, Midgley and co-workers presented a child-adapted model of MBT, named Mentalization-Based Treatment for Children (MBT-C; Midgley et al., 2017). MBT-C is a short-term intervention for children aged 5–12 years, integrating parental involvement to enhance treatment outcomes. Sessions for both children and parents are distinguished by an emphasis on mental states and subjective processes, coupled with an active and playful therapeutic stance.

Given that mentalization-based interventions for children in middle childhood represent a relatively new field, only a limited number of evaluation studies have been conducted to date. In a systematic, narrative review of mentalization-based interventions for children, Midgley, Sprecher et al. (2021b) identified a broad range of mentalization-influenced adaptions, indicating a wide applicability of mentalizing approaches. However, they found only 20 papers reporting outcomes of direct therapeutic work with children and their families, and primarily in the context of adoption or foster care. Many of the studies were of poor quality, frequently lacking comparison conditions, sufficient power and assessment of well-being reported by the children themselves. An intriguing finding was that child-reported outcomes demonstrated greater effects than parent-reported outcomes, underscoring the importance of including children’s subjective reports in future studies on mentalization-based interventions for children. The overview concluded that the evidence-base for MBT-C remains significantly underdeveloped and highlighted the pressing need for further research on short-term interventions targeting mentalizing capacity in middle-childhood disorders (Midgley, Sprecher et al., 2021b).

The present pilot quasi-randomized controlled trial aims to evaluate the feasibility and preliminary effectiveness of short-term mentalization-based therapy for common childhood disorders. By addressing the gaps in current research and focusing on real-world applicability, this study seeks to contribute to the growing body of evidence supporting mentalization-based interventions for young children.

## Method

### Design

The study was designed as a partly controlled effectiveness study in a naturalistic setting, including a wait-list comparison and two follow-ups. The allocation of patients to the treatment group or the wait-list group was quasi-randomised and strictly determined by the time of admission due to the following principles: If less than eight weeks before the summer or Christmas break at the treatment clinic, the patient was allocated to the wait-list. If eight weeks or more, the patient was allocated to the treatment group. Patients allocated to the wait-list were crossed-over to treatment after a period of 12 weeks. Follow-up assessments including all measures were administered at 6 and 12 months after the end of therapy. The study was approved by the regional ethic board (2009/524-31) at the Karolinska Institute, Stockholm.

### Participants

The sample consisted of 17 children, aged 4–11 years. During the two-year inclusion period, their parents had requested psychotherapeutic treatment at the Erica Foundation Clinic for various psychological problems. For inclusion, the child was required to fulfil criteria for one or more psychiatric disorder according to the DSM-IV-TR (American Psychiatric Association, 2002), and present with a significantly impaired global function (i.e., CGAS below 70). Due to the emotional demands imposed in focused, short-term psychotherapy, patients with intellectual developmental disorder, autism spectrum disorder, eating disorder or severe attachment-related issues were excluded from the project. Furthermore, patients expressing substantial suicidality (meaning plans or intent) were excluded. Patients who were excluded were offered an alternative intervention at the clinic. Children and parents who after three assessment sessions were considered to fulfil all inclusion criteria and none of the exclusion criteria were invited to participate in the study.

Once informed consent was collected, depending on the time of admission, the patient was allocated either to MBT-C or a 12-week waiting period, after which MBT-C was offered. Therapists were assigned according to a “next available” principle.

### Assessment

Assessments were conducted at four time points: baseline (after three assessment sessions), end of therapy (12 weeks), and six and twelve months after completing treatment. The wait-list group was assessed at an additional measure point twelve weeks after baseline, i.e., prior to initiating treatment. Participants originally assigned to the control group, when crossed over to treatment, were also followed up at 6 and 12 months post-treatment.

All assessments were based on questionnaires, parent-ratings and medical records from the therapists’ intake interviews. Psychiatric diagnoses according to Diagnostic and Statistical Manual of Mental Disorders fourth ed Text Revision (DSM-IV-TR; American Psychiatric Association, 2002) and the HoNOSCA (Health of the Nation Outcome Scales for Children and Adolescents; Gowers et al., 2000) was conducted by an independent psychiatrist specialized in child and adolescent psychiatry. This assessment was based on all the collected data, such as ratings, medical records and interviews with the therapists. Ratings of global psychic function (CGAS) were conducted by the treating therapists.

#### The health of the nation outcome scales for children and adolescents (HoNOSCA)

The HoNOSCA is a clinician-rated measure developed for child and adolescent mental health services. Several studies have concluded its validity, reliability, and sensitivity as an outcome measure (Garralda et al., 2000; Gowers et al., 2000; Pirkis et al., 2005). The HoNOSCA estimates 13 clinical features on a 0–4 scale with the aim of providing a global score (range 0–52) of the young person’s mental health problems.

#### Children’s global assessment scale (CGAS)

The CGAS is a clinician-rated global measure for assessing the severity of psychiatric problems in children and youngsters (4–20 years). It has a range of 1–100 and provides anchor point descriptions for behavioural function at home, at school, and with peers. Due to its favourable psychometric properties the CGAS is widely used in clinic and research (Dyrborg et al., 2000; Schaffer et al., 1983). A validated Swedish version of the CGAS was used (Lundh et al., 2010).

#### Strengths and difficulties questionnaire (SDQ)

The SDQ (Goodman, 1999, 2001) is a 25-items questionnaire for children 3–16 years of age, filled in by parent and teacher. It is found to yield valid and reliable scores on internalizing symptoms, conduct problems, hyperactivity/inattention, and peer problems. A total difficulties score is calculated by summing four problem scales. The extended version of the SDQ was used, including an impact supplement about overall distress, social impairment, burden and chronicity. In cases where two care takers filled in the SDQ, an average score was calculated and used in the analysis. The Swedish translation of the instrument has shown validity (Malmberg et al., 2003) and to discriminate well between Swedish community and psychiatric samples (Smedje et al., 1999).

#### Child self-rating (“the tell tube”)

Since there, to our knowledge, were no available self-rating questionnaires for younger children, an unvalidated instrument was used. The instrument was developed for clinical purposes but has been tried out in an earlier study on children’s own perspectives on psychotherapy (Carlberg et al., 2009). Even pre-school and early school aged children have been shown to easily understand the instrument and use it for nuanced communication of own feelings and opinions on subjects concerning problems, expectations and experiences related to psychotherapy. The instrument consisted of a transparent plastic tube that children filled with 1–5 paper pulp balls decorated with smileys, indicating their perceived level of emotional distress related to their presenting problems. The instrument was administered pre- and post-therapy as well as at follow-ups.

### Therapists

Four licensed psychotherapists were engaged in the study, two women and two men. Mean age was 54 years (range 45–63). All of them had a Master’s degree in psychology and certified degrees in psychodynamic child and adolescent psychotherapy. They had, on average, 12 years (SD 6.15) of clinical experience of psychodynamic psychotherapy with children. Each therapist treated 4–8 patients. They were alternately engaged in either child or parental work. For fidelity purposes they participated in a weekly peer supervision group. All received additional supervision when needed.

### Treatment characteristics

The treatment was a focused 12-session therapy with parallel parent sessions (ratio 1:1), conducted once weekly, preceded by three assessment sessions. The treatment has been described in Midgley et al. (2017).

MBT follows the principles of play in psychodynamic child therapy, where play is seen as a vehicle for therapeutic communication building on the assumption that children will use play materials to directly or symbolically communicate and process emotions, thoughts, and experiences that they are not able to meaningfully express through words. The therapeutic stance was active and child-centred, searching for a comprehensive understanding of the child’s subjective world and mental representations of interpersonal relationships. Affective and symbolic communication in play and dialogues was encouraged. The therapeutic technique aimed at directing attention to and maintaining the individually negotiated therapeutic focus, while exploring and sharing new intersubjective experiences, and at the same time promoting enhanced emotion regulation and mentalization in the child.

Central working tools in the model were the time-limitation, visualized by a child-drawn calendar where each session was numbered and illustrated, as well as the child-centred formulation of a therapeutic focus. The therapeutic technique aimed at directing attention to and maintaining the negotiated focus, while exploring and sharing new intersubjective experiences, and at the same time promoting an enhanced mentalization and emotion regulation in the child.

The parallel parental sessions with a co-therapist played an essential part in the therapeutic model. These had the same number and frequency as the child’s therapy. Further, the parental work was guided by the same focus formulation as the child’s. The rationale was to enhance parental mentalizing in order to enhance parents’ capacity to reflect on the child’s experiential world and self-representations, as well as on themselves as parents.

### Data analysis

Data analysis was conducted using SPSS, v29.0, unless otherwise stated.

An intent-to-treat (ITT) sample was used. All 17 children completed the treatment. When two parents had answered the SDQ, the mean score of the parent ratings was used.

For the analysis of group-differences between the treatment and wait-list group at post-treatment, outcomes were assessed using ANCOVAs, with the baseline value of the respective measure as covariates. Missing data was only observed on secondary outcome measures, where two observations were missing on the SDQ total and SDQ impact score. Due to the small sample size, missing data was handled with the Last-Observation-Carried-Forward method (LOCF). To test the influence of missing data, we also conducted a multiple imputation as a sensitivity analysis, even though there is little research on performance of multiple imputation in such small samples (e.g., Barnes et al., 2006). Results were pooled over fifty imputed datasets (Jakobsen et al., 2017). Multiple imputation was conducted in R, version 4.2 (The R Foundation), packages Mice (Buuren & Groothuis-Oudshoorn, 2011) and Miceadds (Robitzsch & Grund, 2023). These imputations did not alter the results in any meaningful way. As a result, analyses using LOCF were retained. Results from the sensitivity analysis is presented in the supplemental material. Longitudinal within-group effects and follow-up results were assessed using data from all participants who received treatment. This means that data from participants originally assigned to the control group were also included when they had been crossed over to treatment. Analyses were conducted using piecewise linear mixed models, with the treatment phase (pre-treatment to post-treatment) as piece one, and the follow-up phase (post-treatment to follow-up 2) as piece two. Piecewise modelling of growth trajectories is suitable when there are different phases in the growth model with distinct shapes or trajectories (Heck et al., 2014). This is a reasonable assumption in this case, where there is a treatment phase and a follow-up phase, and a steeper trajectory of change would be expected during the active treatment phase. Models were specified with time on the within-level. For Piece 1, time was coded 0 3 3 3; for Piece 2, time was coded 0 0 6 12. Here, the coding of time represents months, meaning that 3 represents 3 months from baseline (i.e., end of treatment). In Piece 2, the baseline is the post-treatment measurement, represented by 0, and 6 and 12 represents months since end of treatment. This means that the pre-treatment score was coded as the intercept for Piece 1, whilst the post-treatment score was coded as the intercept for Piece 2. Random intercept and slopes were retained when they contributed to the model, as indicated by a lowered AIC of ≥2 indicating superior fit (Burnham & Anderson, 2004). Random effects were assumed independent with different variances. Missing data was handled with the Restricted Maximum Likelihood method.

## Results

### Sample characteristics

Descriptive statistics for the sample are presented in Table 1. The participants were 4–11 years of age with a mean age of 7 (*SD* = 1.66). Out of 17 participants, 15 (88%) reported psychosocial and environmental factors contributing to the psychiatric disorder according to DSM-IV-TR Axis IV. The most frequent environmental factors reported were relational problems between adults in the family (52.9%), followed by relational problems with peers (35.3%), death of a family member (29.4%) and psychiatric health problems in the family (29.4%).

**Table 1.** Sample characteristics pre-treatment (*n* = 17).

	Treatment group (*n* = 10)	Control group (*n* = 7)	Total (*n* = 17)
Mean age (years)	6.40	7.86	7.00
Gender			
Female	6 (60%)	4 (57.1%)	10 (58.8%)
Male	4 (40%)	3 (42.9%)	7 (41.2%)
Main disorder			
Anxiety disorder	2 (20%)	6 (85.7%)	8 (47.1%)
Affective disorder	2 (20%)	0	2 (11.8%)
Disruptive or conduct disorder	4 (40%)	1 (14.3%)	5 (29.4%)
Attention deficit/hyperactivity	1 (10%)	0	1 (5.9%)
Other	1 (10%)	0	1 (5.9%)
Comorbid disorders			
Affective disorder	0	1 (14.3%)	1 (5.9%)
Disruptive/conduct disorder	1 (10%)	1 (14.3%)	2 (11.8%)
ADHD	1 (10%)	1 (13.3%)	2 (11.8%)
Other	4 (40%)	4 (57.2%)	8 (47.1%)

*Note.* Some patients had several comorbid disorders, which is why the total percent can go above 100. Other = problems parent-child, enuresis, encopresis.

Treatment conditions (experimental vs. waiting list) were compared on demographic characteristics (age, gender, number of psychosocial and environmental problems) as well as baseline scores on all outcome measures. Chi-square test for independence revealed that the proportion of participants with an anxiety disorder was significantly larger in the waiting-list control group (85.7%) than in the experimental group (20.0%) [χ^2^ (1, *n* = 17) = 4.74, *p* = .015]. No other differences were found between the conditions.

### Differential treatment effects

**Figure 1.**
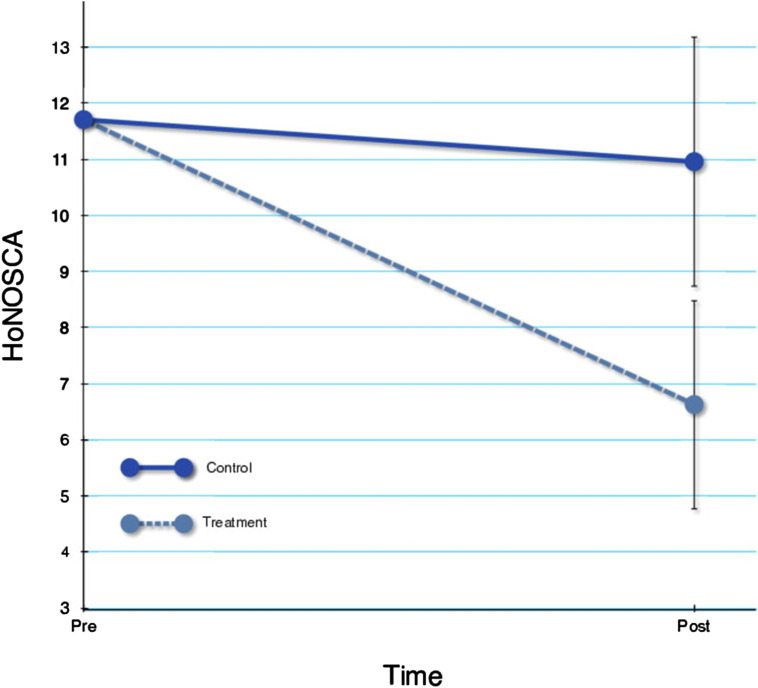
Change over time in HoNOSCA.

For the primary outcome HoNOSCA, the treatment group was significantly superior (F_1,14_ = 10.28, *p* = .006). The estimated difference in raw scores was 4.34, corresponding to an effect size of *d* = 1.23 (95% CI 0.17, 2.29). Results for HoNOSCA are visualized in Figure 1.

For SDQ total score, there was no significant difference between groups (F_1,14_ = 1.27, *p* = .28). The estimated difference in raw scores was 1.94, corresponding to an effect size of *d* = 0.41 (95% CI −0.59, 1.41). However, for SDQ impact score, the treatment group was significantly superior (F_1,14_ = 7.28, *p* = .017). The estimated difference in raw scores was −2.36, corresponding to an effect size of *d* = 1.42 (95% CI 0.34, 2.50). For CGAS, the treatment group was significantly superior (F_1,14_ = 14.08, *p* = .002). The estimated difference in raw scores was 15.07, corresponding to an effect size of *d* = 1.73 (95% CI 0.60, 2.86).

For child-rated problems, again, the treatment group showed significant improvements compared to the waitlist group (F_1,14_ = 6.38, *p* = .024). The estimated difference in raw scores was 1.78, corresponding to an effect size of *d* = 1.32 (95% CI 0.26, 2.38).

### Within-group changes over time

For HoNOSCA, the best model fit was achieved by adding random intercept and random slopes for piece 1 (i.e., the treatment phase) but not piece 2 (i.e., the follow-up phase). Participants improved significantly during treatment (F_1,26_ = 41.52, SE = 0.28, *p* < .001) and continued to improve during the follow-up period (F_1,33_ = 9.18, SE = 0.06, *p* = .005). For SDQ total score, the best model fit was achieved by adding a random intercept but no random slopes. Participants improved significantly from pre- to post-treatment period (F_1,44_ = 6.31, SE = 0.33, *p* = .016). During the follow-up period no further improvements were observed (F_1,43_ = 0.19, SE = 0.09, *p* = .668). For SDQ Impact score, the best model fit was achieved by adding a random intercept but no random slopes. During treatment participants improved significantly (F_1,45_ = 12.71, SE = 0.16, *p* < .001). No significant change was observed during the follow-up period (F_1,43_ = 0.66, SE = 0.04, *p =* .422). For CGAS, the best model fit was achieved by adding a random intercept and random slopes for piece 1 and piece 2. Patients improved significantly on CGAS during treatment (F_1,17_ = 55.54, SE = 0.68, *p <* .001) and during the follow-up period (F_1,15_ = 7.62, SE = 0.15, *p =* .014). For child-rated problems, the best model fit was achieved using random intercept but no random slopes. From pre- to post-treatment participants improved significantly (F_1,45_ = 40.91, SE = 0.13, *p* < .001). No significant change was observed during the follow-up period (F_1,46_ = 0.00, SE = 0.04, *p* = .98). Observed scores and number of observations for each timepoint are presented in Table 2. Slope estimates, estimated change scores and effect sizes are presented in Table 3.

**Table 2.** Observed scores across time points for all participants receiving MBT-C.

	Pre-treatment	Post-treatment	Follow-up 6 mo	Follow-up 12 mo
CGAS				
*M*	52.24	67.0	70.31	71.81
*SD*	5.17	9.42	9.87	10.91
*n*	17	17	16	16
SDQ total score				
*M*	12.38	10.17	9.17	9.32
*SD*	4.64	4.78	5.15	5.17
*n*	17	15	15	14
SDQ impact				
*M*	2.88	1.2	1.27	1.54
*SD*	1.93	1.68	1.71	1.79
*n*	17	15	15	14
HoNOSCA				
*M*	11.35	6.12	4.44	4.20
*SD*	3.06	3.69	3.78	3.97
*n*	17	17	16	15
Child-rated problems^a^				
*M*	1.35	3.75	4.0	3.79
*SD*	1.41	1.44	1.59	1.05
*n*	17	16	14	14

*Note.* CGAS: Children’s Global Assessment Scale; SDQ: Strength and Difficulties Questionnaire; HoNOSCA: the Health of the Nation Outcome Scales for Children and Adolescents.

^a^Higher is better.

**Table 3.** Estimated slopes, change scores and effect sizes during treatment and follow-up.

	Piece 1 estimate	Pre-post change (raw scores, Cohen’s d)	Piece 2 estimate	Change during follow-up (raw scores, Cohen’s d)
HoNOSCA	−1.82	5.46, *d* = 1.78 (95% CI 1.21, 2.35)	−0.17	2.00, *d* = 0.65 (95% CI 0.22, 1.10)
SDQ total	−0.82	2.46, *d* = 0.53 (95% CI 0.10, 0.96)	−0.04	0.46, *d* = 0.10 (95% CI −0.36, 0.56)
SDQ impact	−0.57	1.7, *d* = 0.88 (95% CI 0.38, 1.38)	0.04	-0.42 *d* = −0.22 (95% CI −0.76, 0.32)
CGAS	5.03	15.08 *d* = 2.92 (95% CI 2.09, 3.74)	0.42	5.04 *d* = 0.97 (95% CI 0.22, 1,72)
Child-rated problems	0.83	2.49 *d* = 1.77 (95% CI 1.21, 2.32)	−0.001	−0.01 *d* = −0.01 (95% CI −0.62, 0.61)

*Note.* CGAS: Children’s Global Assessment Scale; SDQ: Strength and Difficulties Questionnaire; HoNOSCA: the Health of the Nation Outcome Scales for Children and Adolescents.

## Discussion

This quasi-randomised naturalistic study examined preliminary effects of MBT-C for children with mixed psychiatric disorders. Treatment showed significant superiority over wait-list on therapist-rated global function and symptomatology as well as child-rated experience of problems and parent-rated overall distress and impairment in the child’s daily life, but not on parent-rated problems. Using all available data (*n* = 17), within-group effects were moderate to large on all outcome measures. Importantly, longitudinal results showed improved gains over a 12-months follow-up for HoNOSCA and CGAS, and maintained gains for SDQ total, SDQ impact and child-rated problems.

The fact that results rated by both therapist, parent and child point in the same direction, indicates robustness of the findings. Interestingly, even though the parent-rated total score of the SDQ did not change significantly, the Impact score, indicating the aggregated burden of the child’s problems on their everyday life, decreased significantly. It is possible that the SDQ is not an optimal instrument to assess psychiatric problems, as it is not symptom oriented.

The promising results during treatment as well as the continuous improvement found for some of the measured during the follow-up period (also known as ‘sleeper effects’) are in line with previous findings for STPP for children and adolescents (Abbass et al., 2013). This is particularly promising seeing as this is a relatively short treatment. Not many studies compare effects of STPP to wait-list controls, but Sinha and Kapur (1999) found an effect size of *g* = 1.42 compared to wait-list, investigating STPP for mixed disorders in adolescent boys. The results from the present study are in line with these previous results.

There is a vast need for studies investigating psychodynamic treatments for children and adolescents. To date, there are a few studies supporting the effects for PDT in emotional disorders, whilst there is a lack of studies specifically targeting anxiety disorders, disruptive behaviour disorders or personality disorders (Midgley et al., 2021a). MBT-C is transdiagnostic, and the present trial includes children with both anxiety disorders and disruptive behaviour disorders, indicating that the treatment may be effective for a wide group of patients. Further research should investigate results for MBT-C for specific diagnostic group, using specific symptom rating scales.

The biggest limitation of the study is the small sample size (*n* = 17). The results should therefore be seen as tentative and preliminary. There was considerable heterogeneity in the results between patients, meaning that a larger patient group is needed in order to obtain more stable results and a more nuanced picture of the efficacy of the treatment. Another limitation is that the groups are not truly randomised, but from different time-frames, opening up for a risk of systematic differences between the groups, hampering the internal validity of the trial. For example, there was a higher rate of anxiety disorders in the wait-list group. We cannot determine if and how this may have influenced the results. On the other hand, clinical ratings of overall functioning according to CGAS did not differ between groups at baseline. The small sample size also prevented us from conducting any analyses of possible moderators or mediators, such as evaluating whether there were specific diagnostic groups that benefitted more from the treatment.

Another limitation is the fact that global functioning (CGAS) was rated by the therapists. Hence, this rating was not blinded and might be inflated. To compensate for this limitation, an independent psychiatrist specialized in child and adolescent psychiatry was engaged to conduct ratings according to HoNOSCA, a structured and valid assessment method.

A strength of the study is the fact that instruments measuring the perspective of both the therapist, the parents and the child were used. One problem in psychological research on early school-age children is that the perspective of the child itself is often lacking, not the least in studies of mentalization-based interventions, as was problematized in the review by Midgley, Sprecher et al. (2021b). There is an absence of self-report measures for children younger than 9–12 years. Even though the tell tube is not a validated instrument, it was a way to get the perspective of the child. Notably, the results of the child-rated outcomes are in line with the ones rated by the parents and the therapists. No manual for the treatment was used, which can be seen as a limitation of the study. The treatment model used was in development during the study, meaning that it had the core principles for mentalization work that all therapists followed, but that the MBT-C model has evolved during and since the study.

Sessions were not video- or audio recorded for adherence controls. Instead, all therapists were affiliated to regular, weekly intervention fidelity groups.

In line with the findings by Midgley, Sprecher et al. (2021b), the key priority for future research on MBT-C for young and middle-childhood ages, is to develop valid measures for assessing child mentalizing capacity, allowing for more targeted studies of MBT-C effectiveness.

### Conclusions

The results of this trial show promise for MBT-C for young children. Results indicated improvements on most measures, and maintained or improved gains during follow-up. This is a small pilot trial, using quasi-randomization, meaning that results need to be replicated in higher-quality trials.

Significant improvements were shown in child pathology (DSM-IV-TR), global psychic function (CGAS), parent-perceived symptoms and their impact on daily life (SDQ), and child-perceived problem severity (The Tell Tube). The positive outcome persisted at follow-ups.

## Supplemental Material

Supplemental Material - Short-term mentalization-based therapy for common childhood mental disorders – a pilot quasi-randomised controlled trial Supplemental Material for Short-term mentalization-based therapy for common childhood mental disorders – a pilot quasi-randomised controlled trial by Agneta Thorén, Karin Lindqvist, Julia Pertoft Nemirovski and Jakob Mechler in Clinical Child Psychology and Psychiatry
