# Supplementary material for: Short-term mentalization-based therapy for common childhood mental disorders – a pilot quasi-randomised controlled trial
Source: Clin Child Psychol Psychiatry. 2025 Jan 27;30(2):436–51. doi: 10.1177/13591045251316619 (PMC11951363; doi:10.1177/13591045251316619)
Supplement: Supplemental Material - Short-term mentalization-based therapy for common childhood mental disorders – a pilot quasi-randomised controlled trial [file sj-pdf-1-ccp-10.1177_13591045251316619.pdf]

Supplementary Table 1. AIC scores for different models tested.

	**HoNOSCA**	**SDQ Total**	**SDQ Impact**	**CGAS**	**Child-rated problems**
Null model w random intercept	379.80	343.50	247.26	510.13	254.18
Random intercept, fixed effects of time, no random slopes	326.51	**337.38**	**240.83**	445.80	**223.37**
Random intercept, fixed effects of time, random piece 1	**321.10**	338.54	242.10	425.60	223.65
Random intercept, fixed effects of time, random piece 2	324.31	338.16	241.15	440.73	225.37 (no convergence)
Random intercept, fixed effects of time, random piece 1 and 2	320.02	338.91	243.02	**418.03**	225.65 (no convercence)

Note. CGAS = Children's global assessment scale; SDQ = Strength and difficulties questionnaire; HoNOSCA = The Health of the Nation Outcome Scales for Children. Bold text indicates the final model chosen.

Supplementary Table 2. Results from ANCOVA after multiple imputation

	**Estimated difference between groups**	**SE**	**p**
Impact	2.12	0.63	0.02
SDQ Total	1.59	2.21	0.49

Note: When pooling estimates based on multiple imputation using the MICE package, an F value is not provided.
